# Role of Tumor Necrosis Factor Superfamily in Neuroinflammation and Autoimmunity

**DOI:** 10.3389/fimmu.2015.00364

**Published:** 2015-07-20

**Authors:** Sandip Sonar, Girdhari Lal

**Affiliations:** ^1^National Centre for Cell Science, Pune, India

**Keywords:** autoimmunity, blood–brain barrier, multiple sclerosis, tumor necrosis factor, neuroinflammation

## Abstract

Tumor necrosis factor superfamily (TNFSF) molecules play an important role in the activation, proliferation, differentiation, and migration of immune cells into the central nervous system (CNS). Several TNF superfamily molecules are known to control alloimmunity, autoimmunity, and immunity. Development of transgenic and gene knockout animals, and monoclonal antibodies against TNFSF molecules have increased our understanding of individual receptor–ligand interactions, and their intracellular signaling during homeostasis and neuroinflammation. A strong clinical association has been observed between TNFSF members and CNS autoimmunity such as multiple sclerosis and also in its animal model experimental autoimmune encephalomyelitis. Therefore, they are promising targets for alternative therapeutic options to control autoimmunity. Although, TNFSF ligands are widely distributed and have diverse functions, we have restricted the discussions in this review to TNFSF receptor–ligand interactions and their role in the pathogenesis of neuroinflammation and CNS autoimmunity.

## Introduction

CD4^+^ T cells are one of the key adaptive immune cells that play an important role in several autoimmune diseases like multiple sclerosis (MS), experimental autoimmune encephalomyelitis (EAE), inflammatory bowel disease (IBD), and collagen-induced arthritis (CIA). Interactions between tumor necrosis factor superfamily (TNFSF) ligands and TNF receptor superfamily (TNFRSF) receptors provide the costimulatory signals that control the survival, proliferation, differentiation, and effector function of immune cells. Therefore, signaling from these ligand–receptor pairs effectively helps in maintaining immune cell homeostasis and in regulating the pathology of autoimmune diseases (1–4).

**Figure 1:**
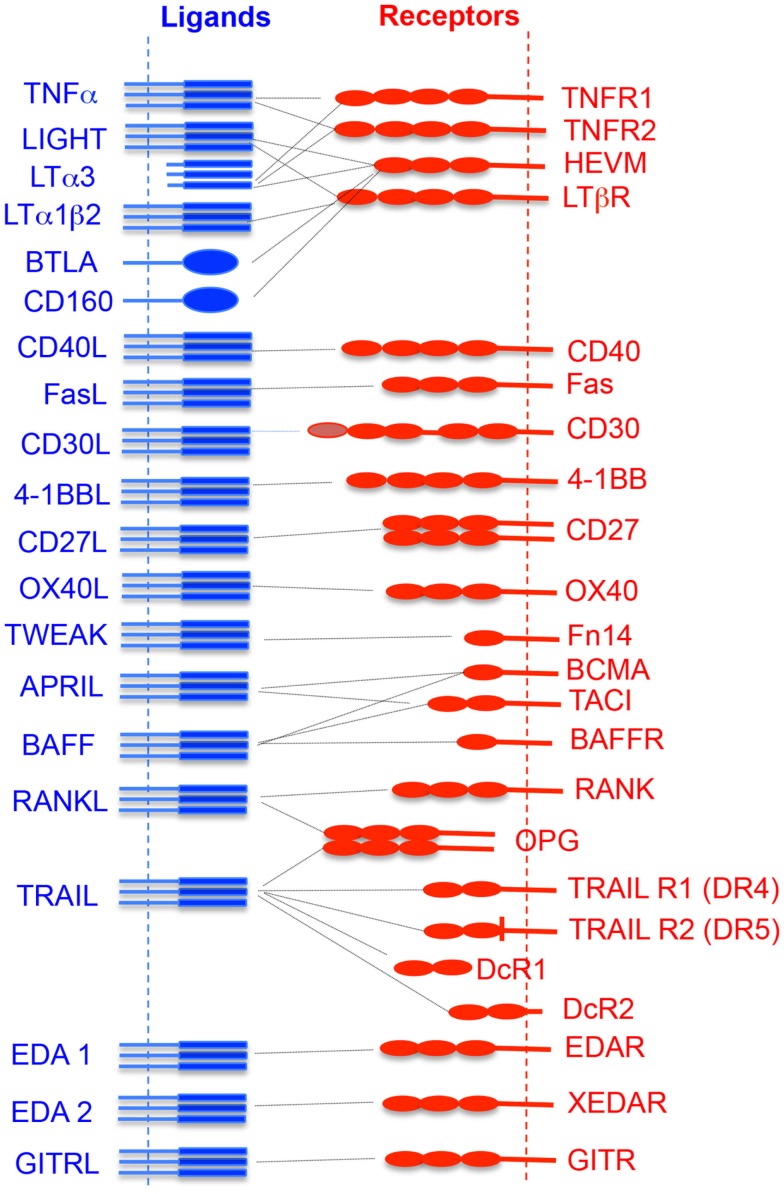
**Interaction of TNF superfamily ligand and its receptors**. The vertical dotted lines represent the cytoplasmic membrane where receptors or ligands are attached. The horizontal dotted line shows the receptor and its cognate ligand interaction.

About 19 TNFSF ligands have been identified, which include TNF-α, TNF-β [also known as lymphotoxin alpha (LTα)], lymphotoxin-β (LT-β), CD27L, CD30L, CD40L, FasL, 4-1BBL, OX40L, TNF-related apoptosis-inducing ligand (TRAIL), LIGHT (homologous to lymphotoxins, exhibits inducible expression, and competes with HSV glycoprotein D for herpesvirus entry mediator (HVEM), a receptor expressed by T lymphocytes), receptor activator of NF-κB ligand (RANKL), TNF-related weak inducer of apoptosis (TWEAK), a proliferation-inducing ligand (APRIL), B-cell activating factor (BAFF), vascular endothelial cell-growth inhibitor (VEGI), ectodysplasin A (EDA-A1, EDA-A2), and glucocorticoid-induced TNFR family-related gene ligand (GITRL) (Figure 1). While expressions of TNFSF ligands are induced largely on professional antigen-presenting cells (APCs; dendritic cells, B cells, macrophages), their expression is also reported on T cells, NK cells, mast cells, eosinophils, basophils, endothelial cells, thymic epithelial cells, and smooth muscle cells (5).

TNF receptor superfamily members are transmembrane proteins having cysteine-rich motifs in their extracellular domains that bind to their cognate ligands (Figure 1). About 30 members of TNFRSF have been identified (3, 6, 7). Depending upon the specific intracellular signal induced by TNFRSF members, they can be categorized into three groups – death domain (DD)-containing receptors, decoy receptors, and TNF receptor-associated factor (TRAF)-binding receptors (8). Some TNFRSFs such as TNFR-1, Fas, DR3, DR4, DR5, and DR6, contain their own DDs. However, they also interact with other cytoplasmic DD-containing adaptor molecules (9). This receptor–adaptor complex acts as a scaffold for binding of immature pro-caspase, which then undergoes auto-cleavage, leading to the formation of the death-inducing signaling complex (DISC) and induction of apoptosis (9, 10). Some other TNFRSFs, such as TNFR-2, CD27, CD30, CD40, glucocorticoid-induced TNFR family-related gene (GITR), Fn1, lymphotoxin beta-receptor (LTβR), OX40, receptor activator of NF-κB (RANK), and XEDAR, lack a DD and contain motifs with four to six amino acids called TRAF-interacting motifs (TIMs) which recruits TRAF proteins. TRAF proteins are adaptor molecules that activate multiple downstream signaling pathways such as NF-κB, Janus kinase (JNK), ERK, p38MAPK, and PI3K that help in cell survival, proliferation, and cytokine production (11).

There are at least five anti-TNF medications (Etanercept, Infliximab, Adalimumab, Golimumab, and Certolizumab) approved by the U.S. Food and Drug Administration (FDA) for the treatment of rheumatoid arthritis (RA). Most of these biologics bind to soluble TNF molecules and prevent their binding to TNF-receptors. This blocks the production of pro-inflammatory cytokines such as IL-1, IL-6, or IFN-γ. Anti-TNF antibodies can also bind to surface-expressed TNF molecules and induce reverse signaling or antibody-induced cell death (AICD) (12, 13). In this review, we discuss the role of TNFSF–TNFRSF members that play a role in neuronal inflammation, the possible molecular mechanisms involved, and the efficiency of these molecules in controlling central nervous system (CNS) inflammation and autoimmunity.

The CNS is considered as an immune-privileged site and consists of a network of CNS microvessels. These microvessels are formed by a highly specialized endothelial lining supported by astrocytes, pericytes, microglial cells, and neurons, which together form a very firm blood–brain barrier (BBB). The BBB restricts entry of immune cells into the CNS, thereby actively maintaining a homeostasis. However, under inflammatory conditions, the BBB gets disrupted and immune cells migrate into the CNS parenchyma. A disrupted BBB is one of the hallmarks of autoimmune demyelinating diseases like MS and EAE (14–16).

Watts et al. have reported that vascular endothelial growth factor receptor (VEGFR) signaling activates JNK and positively regulates the angiogenesis and barrier property of BBB endothelial cells (17). They also reported that death receptor 6 (TNFRSF21) and TROY (TNFRSF19) were regulated in the acquisition and development of barrier property in BBB (17). Both these TNFRSF members are downstream targets of the Wnt/beta-catenin signaling pathway in the BBB endothelial cells. Dysregulation of TNFRSF21/TNFRSF19 signaling leads to the disruption of endothelial BBB. Since Wnt/beta-catenin signaling is required for CNS angiogenesis but not for peripheral vasculature (18, 19), an understanding of the molecular mechanism of this signaling would help in designing novel therapeutics or biologics that target TNFSF–TNFRSF interactions, to control CNS autoimmunity.

**Figure 2:**
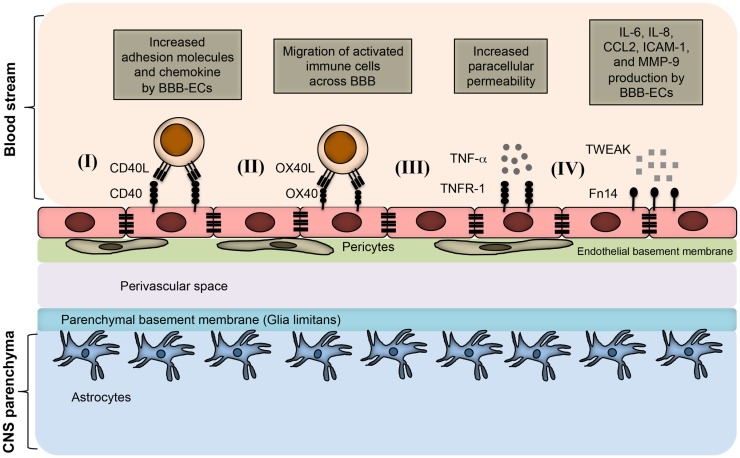
**TNFSF receptor–ligand interaction at endothelial BBB during neuroinflammation**. BBB endothelial cells express TNFSF receptors during inflammatory condition, and interact with the TNFSF ligand in soluble form as well as on infiltrating immune cells. (I) Inflamed BBB endothelial cells express CD40. Interaction of CD40 with CD40L-expressing activated immune cells leads to up-regulation of adhesion molecules and chemokine secretion by BBB endothelial cells. This promotes the migration of pathogenic immune cell subsets into the CNS parenchyma. (II) OX40 expression can be induced in BBB endothelial cells during inflammation, which facilitates the migration of OX40L^+^ immune cells across the BBB. (III) Under inflammatory conditions, BBB endothelial cells up-regulate TNFR-1, which bind to soluble TNF secreted from various immune cells, such as activated Th1 cells, B cells, macrophages, and NK cells. Binding of TNF with TNFR-1 increases the paracellular permeability of BBB endothelial vessels. (IV) Inflamed BBB endothelial cells express Fn14 that binds to soluble TWEAK molecules. This leads to the up-regulation of cytokines, chemokines, cell adhesion molecules, and matrix metalloprotenase-9 (MMP-9). Increased expression of CCL2 and ICAM-1 facilitates the migration of pathogenic immune cells; whereas MMP-9 helps in the degradation of laminin molecules present in the basement membrane, resulting in loosening of the BBB.

**Figure 3:**
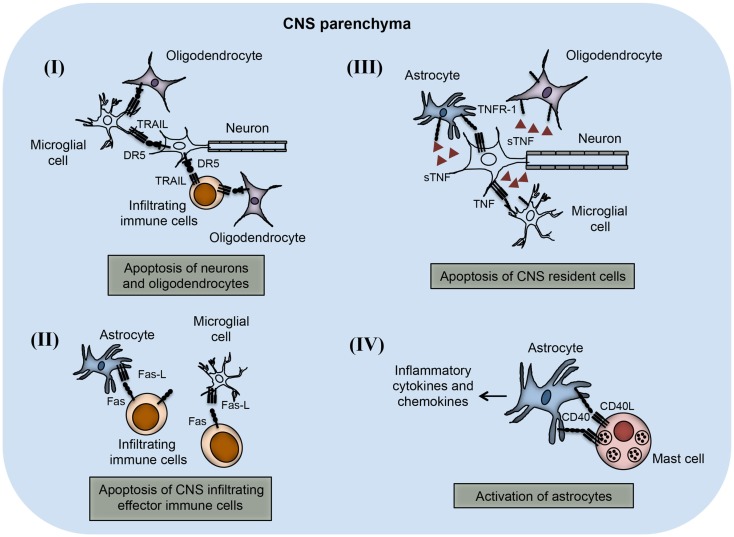
**TNFSF receptor–ligand interaction in the CNS parenchyma during neuroinflammation**. TNFSF receptors and ligands are expressed on both CNS infiltrating effector immune cells and CNS-resident cells. The interaction of this receptor–ligand greatly influences the outcome of neuroinflammatory disease like multiple sclerosis and EAE. (I) Both neurons and oligodendrocytes express functional DR5 in the CNS during EAE. DR5 on the neurons as well as on oligodendrocytes interacts with TRAIL molecules present on either microglial cells or infiltrating immune cells, leading to apoptosis of DR5-expressing cells. (II) Activated astrocytes and microglial cells up-regulate FasL expression on their surface. The interaction of FasL with Fas-expressing cells leads to apoptosis and elimination of pathogenic effector immune cells. (III) Neuronal cells express TNF and that can interact with TNFR-1 present on various CNS-resident cells, such as astrocytes, microglial cells, and oligodendrocytes. Interactions of TNF with TNFR-1-expressing cells lead to apoptosis of TNFR-1^+^ cells. (IV) Mast cells are known to localize close to the astrocytes during EAE in the brain. CD40L present on mast cells interact with CD40-expressing astrocytes, which induces increased production of inflammatory cytokines and chemokines. Local production of inflammatory molecules can augment inflammation and tissue damage in the CNS.

The importance of TNF superfamily receptors and ligands in neuroinflammation are listed in Table 1. Some of the receptor–ligand interactions and their function at BBB (Figure 2) and in the brain parenchyma (Figure 3) are depicted. The role of important TNFSF–TNFRSF pairs in neuroinflammation and autoimmunity are discussed in details below.

**Table 1:** **TNF superfamily in neuroinflammation**.

TNFSF receptor	TNFSF ligand	Blocking/genetic deficiency/over-expression	Effect on neuroinflammation in EAE or MS	Reference
TNFR-1 and TNFR-2	TNFα	Anti-TNFα or TNFR-1-IgG	Reduced demyelination but CNS infiltration is unaffected in EAE	(22)
		TNFα^−/−^	Severe EAE	(25, 26)
		Neuron-specific TNF transgenic mice	Spontaneous EAE	(27)
		TNFR-1/2^−/−^	Mild EAE	(30)
		TNFR-2^−/−^	Hyper susceptible to EAE	(30)
OX40	OX40L	Anti-OX40	Substantial inhibition of EAE	(81)
		OX40-Ig	Mild EAE	(82)
		Anti-OX40L	Reduced infiltration in spinal cord in both active and passive EAE	(84)
		OX40L^−/−^	Controls active EAE, but do not have effect on passive EAE	(138)
4-1BB	4-1BBL	Agonistic anti-4-1BB (2A)	Reduced incidence and severity of EAE. It also prevents the relapse of EAE	(87)
		4-1BBL^−/−^	Controls EAE by reducing VCAM-1 expression on spinal cord endothelial vessels	(89)
GITR	GITRL	GITRL blocked by anti-GITRL antibody on B cells	Severe passive EAE	(100)
DR4/DR5	TRAIL	Recombinant soluble TRAIL	Delayed onset and mild EAE	(102)
		Anti-TRAIL antibody	Severe EAE	(102)
		TRAIL^−/−^	Hypersusceptibility to EAE	(102)
Fn14	TWEAK	TWEAK transgenic mice	Hypersusceptibility to EAE	(109)
		Recombinant soluble TWEAK	Severe demyelination	(110)
		Anti-TWEAK	Mild EAE	(116)
		Fn–TRAIL fusion protein	Delayed onset and mild EAE	(118)
CD40	CD40L	Anti-CD40L	Prevents relapse when administered at acute phase of relapsing–remitting EAE	(137)
			Substantial inhibition of EAE	(135)
		CD40L^−/−^	Prevents EAE	(136)
		CD40^−/−^	Protects from EAE	(138 –140)
Fas	FasL	Fas^−/−^ or FasL^−/−^	Resistant to EAE	(39, 40)
		Fas *(Ipr)* and FasL *(gld)* mutant mice	Spontaneous EAE	(37, 38)
		Recombinant FasL	Reduces the incidence and severity of EAE	(41, 42)
		Astrocyte-specific FasL^−/−^	Reduces the severity of EAE	(45)
CD27	CD70	Anti-CD70	Suppresses EAE	(121)
		CD70^−/−^ or CD27^−/−^	Severe EAE	(124)
		CD70-transgenic mice	Reduced demyelination and mild EAE	(125)
		MOG_35–55_-specific TCR transgenic mice overexpressing CD70 in B cells (2D2 × CD70 Tg)	Increases the incidence and severity of spontaneous as well as MOG_35–55_-induced EAE	(126)
BAFF-R	BAFF	BAFF-R^−/−^	Exacerbation of EAE	(57)
		Soluble human BCMA-Fc	Delays the onset and reduces the severity of human recombinant MOG (MOG_1–121_)-induced EAE in C57BL/6 mice	(59)
		Anti-BLys (Anti-BAFF)	Attenuated EAE in marmoset monkeys	(61)
LTβR	LTα	LTβR–Ig fusion protein	Mild EAE	(128)
		LTα^−/−^ or LTβ^−/−^	Mild EAE	(130)
HVEM	LIGHT, LTα, BTLA, and CD160	LIGHT^−/−^	Severe EAE with high mortality	(131)
		HVEM^−/−^	Hyper-susceptibility to EAE	(141)

## TNFR–TNFα

Tumor necrosis factor alpha (TNFα or TNFSF2) is a homotrimeric transmembrane protein that plays an important role in systemic inflammation. TNFα is expressed as a membrane-bound precursor (tmTNFα), which is later cleaved between Ala^76^–Val^77^ by a metalloproteinase known as TNFα-converting enzyme (TACE), and released as soluble TNFα (sTNFα). TNFα is produced by many cell types, including activated macrophages, dendritic cells, monocytes, NK cells, CD4^+^ T cells, CD8^+^ T cells, astrocytes, and microglia (20). TNFα interacts with two receptors; TNF receptor type-1 (TNFR-1, also known as CD120a, p55/60) and TNF receptor type-2 (TNFR-2, also known as CD120b, p75/80). Low and constitutive expression of TNFR-1 is found on almost all nucleated cells, and can be activated by both membrane-bound TNFα (tmTNFα) and sTNFα. However, expression of TNFR-2 is inducible and restricted to myeloid and lymphoid lineages. TNFα acts as a pro-inflammatory cytokine during the early phase of EAE, but shows immunosuppressive properties in the later phase. To be biologically functional, tmTNFα and sTNFα monomers must form homodimers. While both tmTNFα and sTNFα can bind to TNFR-1, sTNFα interacts with higher affinity than TNFR-2, leading to an inflammatory response. The tmTNFα mainly interacts with TNFR-2 and promotes cell survival (21). Blocking of TNFα by the administration of a soluble TNFR-1–IgG fusion protein or neutralization of anti-TNFα antibody has been shown to control the development of EAE (22). Interestingly, these biologics prevented demyelination but did not control the infiltration of immune cells into the CNS (22). Furthermore, such regimens also reduced the activation of CD4^+^ T cell and microglia cells, and prevented demyelination in CNS (23, 24). While Korner et al. reported that a deficiency of TNFα only delays the disease progression and resulted in disease of comparable severity (25), others have reported that TNFα-deficient mice developed a more severe myelin oligodendrocyte glycoprotein (MOG) peptide-induced EAE as compared to wild-type littermate controls (26). Expression of TNFα, specifically in neurons in the transgenic mice led to the development of spontaneous inflammatory demyelination (27). It has been reported that deficiency of TNFα, LT-α, and TNFR-1 or neutralization of TNF-α and LTα with monoclonal antibodies greatly reduced the severity of EAE (28–30). In contrast, TNFR-2-deficient mice developed more severe inflammation and demyelination in MOG_35–55_-induced EAE (30). Specific expression of tmTNFα in the transgenic mice showed reduced initiation and severity of the EAE, suggesting that selective targeting of sTNFα/TNFR-1 signaling may be helpful in controlling CNS autoimmunity.

In MS patients, TNFα were found to be expressed at very high levels in the CNS lesions and cerebrospinal fluids, but not in serum (23, 24), suggesting that TNFα was produced locally in the inflamed CNS. This indicates that TNFα might be a good target for therapy against MS. However, while TNFα inhibitors showed protection in the mouse model of EAE, adverse effects were observed during clinical trials in MS patients (21). Similarly, a clinical trial with TNFR-1 fusion protein in MS patients showed disease exacerbation, and the trial was stopped (31). This increased demyelination with TNFα antagonist might be due to the several possible reasons such as: (a) down-regulation of anti-inflammatory cytokine IL-10 and increased production of inflammatory cytokine IL-12 and IFN-γ (32, 33); (b) down-regulation of TNFR-2, which is known to regulate the proliferation of oligodendrocytes and damage repair (34); (c) possibility of reduced or non-permeability of the BBBs to TNFα blockers which would prevents their direct action through these molecules could still enhance the disease by increasing auto-reactive T cells in the peripheral tissues, which can migrate into the CNS and damage the tissues (35); (d) unmasking of latent infection, which is critical for inducing the autoimmune demyelination reaction (36). Anti-TNFα therapy has also been used to control other autoimmune diseases such as RA. However, keeping in mind the possibility of demyelination of the central and peripheral neuronal tissues by such therapy, caution must be exercised, and careful monitoring of any pre-existing neuronal disease or its development following treatment with TNFα antagonists would be prudent.

## Fas–FasL

Fas ligand (FasL, also known as CD95L, TNFSF6) is a type II transmembrane protein expressed on a variety of cells, including CD8^+^ T cells and oligodendrocytes. It binds to the Fas receptor (Fas, also known as CD95, TNFRSF6, apoptosis antigen 1) and the decoy receptor 3 (DcR3). FasL–Fas interactions lead to the formation of a DISC in the Fas-expressing cells, leading to induction of apoptosis. The interaction of Fas with FasL on CNS infiltrating cells or activated CNS-resident cells (microglia, astrocytes, and neurons) also leads to the induction of apoptosis. FasL–Fas interactions play a very important role in immune cell homeostasis, and its dysregulation leads to various autoimmune diseases. Mice with autosomal recessive mutations in Fas (lymphoproliferative, *lpr* mice; lack Fas expression) and FasL (generalized lymphoproliferative disease, *gld* mice; lack FasL expression) genes develop a spontaneous autoimmune syndrome; produce autoantibodies and accumulate a large number of CD4^−^CD8^−^ T cells in the secondary lymphoid tissues, leading to the progressive development of lymphadenopathy and splenomegaly (37, 38). Fas and FasL-deficient mice are resistant to MOG-induced EAE, compared to wild-type mice (39, 40), and intrathecal injection of recombinant FasL protein or neutralizing with anti-FasL antibody suppresses acute EAE (41, 42). Adoptive transfer of myelin basic protein (MBP)-specific FasL^−/−^ CD4 T cells in the wild-type host showed reduced EAE pathology suggesting that FasL expression regulates the encephalitogenic function of T cells (43). It would be interesting to study how these auto-reactive CD4^+^ T cells cross BBB and cause neuronal tissue damage. Determining the temporal and site-specific expression of FasL in a variety of CNS-resident cells during ongoing neuroinflammation might provide valuable clues to address this question. It has been also reported that *Lpr* mice on SJL/J background are completely susceptible to the proteolipid protein (PLP_139–51_)-induced EAE (40). This discrepancy might be due to the involvement of other effector pathways mediated by various TNFRSF members in the CNS.

Astrocytes constitutively express FasL, and they show both positive and negative roles in the inflammation and the development of EAE (44). Using targeted deletion of FasL, specifically in astrocytes, Wang et al. showed that astrocytic FasL is required for the elimination of auto-reactive T cells in the CNS (45). Gamma-delta (γδ) T cells play a very important role in EAE (46), and their deficiency results in chronic EAE (47). γδ T cells were shown to regulate CNS inflammation and disease recovery in a FasL–Fas-dependent manner by controlling the encephalitogenic CD4^+^ T cells (47). Collectively, these reports suggest that Fas–FasL-mediated apoptosis acts as an intrinsic regulatory mechanism to control neuroinflammation and development of CNS autoimmunity.

## BAFF-R–BAFF

The B-cell activating factor of the tumor necrosis factor family (BAFF; also known as TNFSF13B, CD257, B lymphocyte stimulator “BLys”), was originally described as a molecule secreted by T cells and dendritic cells that provide maturation and survival signals to peripheral B cells (48). Its expression was also reported in other cell types such as macrophages, dendritic cells, and neutrophils (49). BAFF transgenic mice have elevated numbers of B cells and effector T cells, and show symptoms similar to that seen in B-cell-mediated autoimmune diseases (50). Immature B cells in the bone marrow express BAFF-receptor (BAFF-R), and its expression is also up-regulated during the development from transitional B-cell stage to mature B cell in the secondary lymphoid organs (51, 52). BAFF also interacts with two more known receptors expressed on the B cells; transmembrane activator calcium modulator and cyclophilin ligand interactor (TACI) and B-cell maturation factor (BCMA) (53). TACI is known to be expressed on marginal zone B cells, and BCMA on germinal center B cells, plasma cells, and memory B cells (54). In addition to BAFF, TACI and BCMA are also known to interact with another TNFSF member known as APRIL (55, 56). Genetic deficiency of BAFF-R in mice resulted in hyper-susceptibility to MOG_35–55_ peptide-induced EAE (57), suggesting that this receptor has a regulatory function in EAE. It has been reported that defects in the BAFF–BAFF-R signaling adversely influence the regulatory B-cell (Breg) function, inducing early onset and severe pathology of the disease (58). Huntington et al. showed that administration of hBCMA–Fc fusion protein in C57BL/6 mice not only reduced the B-cell numbers in peripheral blood, lymph nodes, and spleen, and resulted in a reduced titer of MOG-specific antibody in the serum but it also hampered the activation and differentiation of CD4^+^ T cells (59). It has also been reported that BAFF can enhance the auto-reactive Th17 response, leading to increased progression of EAE (60). Antibody-mediated blocking of BAFF with anti-human BLys attenuated the EAE in marmoset monkeys (61). Glatiramer acetate (GA) is the approved frontline drug for the treatment of EAE and MS, which acts on both innate as well as T and B cells during EAE, and induces an anti-inflammatory microenvironment in the neuronal tissue (62–65). GA was also shown to reduce BAFF mRNA expression in the brain, and decrease the number of BAFF-R^+^ B cells, but not the TACI^+^ B cells in the spleen of EAE mice (66). These reports clearly indicate that BAFF–BAFF-R signaling contributes to the pathogenesis of EAE, and its perturbation may provide a valuable therapeutics tool to control CNS autoimmunity.

## OX40–OX40L

The OX40 (also known as ACT35, CD134, TNFRSF4) is a type I glycoprotein of ~50 kD, which is expressed on activated T cells, such as Th1, Th2, Th17, Foxp3^+^ regulatory CD4^+^ T cell (Treg), and CD8^+^ T cells (67). The OX40 ligand (OX40L also known as gp34, CD252, TNFSF4) is a type II glycoprotein expressed on APCs, such as dendritic cells, B cells, macrophages, and endothelial cells (68). The interaction of OX40–OX40L provides a costimulatory signal to T cells, which leads to their activation and cytokine secretion (69). Inhibiting OX40–OX40L interactions protects from many inflammatory autoimmune diseases, including EAE (70, 71). OX40–OX40L signaling in CD4^+^ T cells promotes the expression of IL-12Rβ2 (72) while inhibits the expression of CTLA-4 (73), Foxp3 (74–76), and IL-10 (77). Selective up-regulation of IL-12Rβ2 leads to the differentiation of Th1, whereas loss of Foxp3, CTLA-4, and IL-10 inhibits the suppressive function of Tregs. Thus, the loss of balance between pathogenic and regulatory cells could promote autoimmune pathology. A growing body of evidences suggests that OX40–OX40L interactions regulate the differentiation of CD4^+^ T-cell subsets, and deficiency of OX40 impairs Treg development (78). This might be because the reverse signaling through OX40L in APCs leads to the production of cytokines IL-6 and IL-12, which regulates the differentiation of effector CD4^+^ T cells (68). It has been shown that OX40L–OX40 signaling inhibits Foxp3 expression (75, 79), and acts as a strong differentiating factor for Th9 in airway inflammation (80). These studies suggest that OX40–OX40L signaling could promote inflammatory responses, in addition to suppressing anti-inflammatory responses in CD4^+^ T cells (68, 78). All these observations suggest that functional plasticity of the CD4^+^ T-cell subsets is also controlled by OX40L–OX40 interactions.

OX40–OX40L interactions in CD28-deficient mice initially led to the identification of OX40–OX40L signaling as being costimulatory in EAE, and blocking these interaction-protected mice from EAE (71). In the rat EAE model, pathogenic OX40^+^ CD4^+^ T cells were found in CNS lesions, and neutralization of OX40 by anti-OX40 antibody ameliorated the EAE (81). Targeting OX40–OX40L interaction with an OX40–Ig fusion protein at the onset of EAE greatly reduced the severity of the disease (82). Brain endothelial cells are the central component of the BBB and are known to express OX40L, which recruit OX40^+^ auto-reactive T cells into the CNS (82). OX40 treatment of human umbilical cord endothelial cells (HUVECs) lead to the up-regulation of CCL5, suggesting that this signaling also modulates endothelial cells to attract a selective population of immune cells at the BBB (83). It would be interesting to study whether OX40 signaling affects the expression of cell adhesion molecules, chemokines, and integrins in the brain endothelial cells. Blocking OX40L by anti-OX40L antibody reduces the infiltration of OX40^+^ myelin-specific T cells in the spinal cord without affecting the priming of the myelin-specific CD4^+^ T cells in the draining lymph nodes in active as well as passive EAE (84). Therefore, OX40–OX40L not only controls the activation and differentiation of CD4^+^ T cells, but also potentiates their migration into the CNS. Together, these reports indicate that OX40–OX40L supports the development of neuroinflammation and CNS autoimmunity.

## 4-1BB (CD137) – 4-1BBL (CD137L)

4-1BB (TNFRSF9) is expressed on activated CD4^+^ T cells (Th1, Th2, and Treg), CD8^+^ T cells, B cells, dendritic cells, NK cells, NKT cells, and mast cells, whereas its ligand, 4-1BBL, is expressed on activated APCs (macrophages, B cells, and dendritic cells), CD4^+^ and CD8^+^ T cells, NK cells, mast cells, and smooth muscle cells (85). Under inflammatory condition, neurons are also known to express 4-1BBL (3). B7-deficient APCs deliver the costimulatory signal to CD4^+^ T cells through 4-1BBL in a TRAF2-dependent manner (86). It has been reported that stimulation of human CD8^+^ T cells with 4-1BBL leads to the production of effector molecules such as perforin and granzyme (3). Antibody treatment with agonist anti-4-1BB (2A) in induced EAE resulted in milder disease, but failed to control the development of passive EAE by adoptive transfer of MOG_35–55_-specific CD4^+^ T cells (87). Anti-4-1BB (2A) treatment induced IFN-γ and granulocyte–macrophage colony-stimulating factor (GM-CSF) secretion by MOG_35–55_-specific CD4^+^ T cells, leading to differentiation of inflammatory Th1 (87). It has been suggested that targeting 4-1BB or 4-1BB ligand could lead to the induction of unresponsiveness in the CD4^+^ T cells during EAE (88, 89). 4-1BBL^−/−^ mice showed reduced expression of vascular cell adhesion molecule-1 (VCAM-1) on spinal cord endothelial vessels, which play an important role in the migration of immune cells in the inflamed CNS (89). 4-1BBL downstream signaling induced the production of reactive oxygen species (ROS) in microglia, leading to apoptosis of oligodendrocytes in the EAE (85). Therefore, these studies indicate that 4-1BB–4-1BBL interactions not only promote the activation of T cells, but also control the migration of myelin-specific CD4^+^ T cells in the CNS. Furthermore, 4-1BBL signaling has a destructive role in the inflamed CNS during EAE.

## GITR–GITRL

Glucocorticoid-induced TNFR family-related gene (also known as CD357, TNFRSF18) is expressed at the low levels in resting mouse and human T cells, but is up-regulated on activated CD4^+^ and CD8^+^ T cells. GITR is constitutively expressed on Tregs (90, 91). GITR–GITRL interactions inhibit the suppressive function of CD4^+^CD25^+^ Tregs and therefore, blocking these interactions breaks the peripheral immune tolerance (92). GITRL is expressed at the low levels on B cells, macrophages, bone marrow-derived dendritic cells, and endothelial cells (3). GITRL can act as a costimulatory signal to CD4^+^CD25^−^ and CD4^+^CD25^+^ T cells (93), and to CD8^+^ T cells in the presence of suboptimal concentrations of anti-CD3ε, in the absence of CD28-mediated signaling (94). In addition, signaling through GITR stimulates the production of cytokines such as IFN-γ, IL-2, IL-4, and IL-10 in CD4^+^ T cells (95).

Regulatory B cells are known to produce anti-inflammatory cytokine IL-10 (96) and suppress CNS autoimmunity (97). Transgenic mice constitutively expressing GITRL on B cells (GITRL^+^ B cells) showed a significantly higher numbers of peripheral Tregs, suggesting that GITRL^+^ B cells might play a role in the homeostasis of Tregs (99). Animals that received, B cells pre-treated with anti-mouse GITRL antibody showed significantly decreased Tregs induction and severe EAE, suggesting that GITRL^+^ B cells support the proliferation and homeostasis of Tregs (100). Treatment with Rituximab, a monoclonal antibody that depletes B cells, including Bregs, leads to severe exacerbation of human ulcerative colitis, suggesting that Bregs play an important role in controlling CNS autoimmunity (98). A peripheral increase in Treg frequency can inhibit the proliferation of myelin-specific effector CD4^+^ T-cell subsets in the secondary lymphoid organs, and can therefore control CNS autoimmunity. This clearly indicates that B cells, through the expression of GITRL, promote and maintain the expansion of Tregs and contribute to the maintenance of immune tolerance.

## DR4/5–TRAIL

TNF-related apoptosis-inducing ligand (also known as CD253, TNFSF10) is a type II membrane protein that binds to two death receptors, DR4 (TRAIL-RI) and DR5 (TRAIL-RII). These receptors are known to induce apoptosis in various cell types in a caspase-dependent manner. Both DR4 and DR5 are known to be expressed in humans, whereas mice express only DR5 (101). TRAIL also binds to the decoy receptors, DcR1 (lack cytoplasmic domain) and DcR2 (truncated DD). Rather than inducing apoptosis, binding to these receptors induces NFκB activation. TRAIL receptors are expressed in neurons and oligodendrocytes, but TRAIL is completely undetected in a healthy CNS. Signaling from TRAIL–DR4/5 is implicated in the pathogenesis of MS and EAE (101). TRAIL is generally found on infiltrating immune cells and activated microglia in the MS lesions. The DR4/DR5–TRAIL interaction seems to induce oligodendrocyte and neuronal death during ongoing EAE. TRAIL-induced apoptosis of neurons and oligodendrocytes contribute to the development of brain inflammation. Blocking brain-specific TRAIL leads to a reduction in the severity of EAE. Similarly, therapeutic treatment with soluble TRAIL delays the onset of the disease and reduces its severity (102). TRAIL is also reported to inhibit Th1 response and promote the suppressive function of Tregs (103). Genetically modified dendritic cells (ES-DCs), which can simultaneously present MOG peptides in association with MHC-II and express TRAIL, cause a reduction in the severity of EAE induced by both MOG_35–55_ and MBP (104, 105). One of the mechanisms through which these ES-DCs controls EAE is by promoting the proliferation of CD4^+^CD25^+^ Tregs, which suppress myelin-specific effector CD4^+^ T-cell responses. It might be possible that TRAIL signaling has a dual role; it may promote suppressive phenotype in the secondary lymphoid organs, whereas in local inflamed CNS, it contributes to tissue damage. Therefore, delineating the role of TRAIL signaling in a spatio-temporal manner during the neuroinflammatory events might help in understanding the complexity of CNS autoimmunity. Osteoprotegerin (OPG), a secreted protein under physiological conditions, shows a lower binding affinity for TRAIL (106); however, its role in CNS inflammation and autoimmunity is not well studied. All these studies suggest that, the apoptosis-inducing property of TRAIL in inflamed CNS contributes to the development of inflammation and CNS autoimmunity.

## TWEAK–Fn14

TNF-like weak inducer of apoptosis (TWEAK; TNFSF12) is a pro-inflammatory and pro-angiogenic cytokine synthesized as a type II transmembrane protein. However, it can be cleaved to give rise to soluble cytokine (107). Both membrane-bound and soluble TWEAK bind to the only known receptor, fibroblast growth factor-inducible 14 (Fn14; also known as TNFRSF12A). TWEAK is expressed on monocytes, microglia, and astrocytes in the healthy CNS. Its expression goes up during CNS inflammation. Fn14 is also expressed on CNS-resident cells such as brain endothelial cells, astrocytes, and neurons (108, 109). Over-expression of soluble TWEAK by injecting TWEAK-encoding recombinant plasmid (110), or in TWEAK transgenic animals resulted in increased severity of EAE (109). These studies indicate that local expression of TWEAK and Fn14 in the CNS could be a critical contributing factor to the pathology of neuroinflammation. One of the mechanisms by which TWEAK elevates CNS inflammation is by inducing CCL2 secretion from brain endothelial cells and astrocytes. CCL2 is a potent activator of neuroinflammation, and plays an important role in the pathogenesis of EAE and MS (111, 112). TWEAK–Fn14 signaling in BBB endothelial cells compromises its barrier property in the mouse models of cerebral ischemia. Therefore, it is possible that during neuroinflammation such interactions can affect the BBB, allowing the myelin-specific cells and soluble mediators to enter into the CNS parenchyma (113, 114).

TWEAK–Fn14 binding does not induce ligand-activated kinase signaling due to lack of cytoplasmic DD, but it triggers the engagement of the TRAF, an adaptor protein that activates the ERK1/2, PI3K/Akt, and NF-κB signaling pathways. Membrane-bound TWEAK is a more powerful inducer of the classical NF-κB signaling pathway than its soluble forms (115). It has been shown that inhibition of TWEAK–Fn14 signaling decreases the severity of EAE (110, 116) and CIA (117). Neutralization of TWEAK after the priming phase with monoclonal antibody controls immune cell infiltration into the neuronal tissues and reduce the pathology of EAE (116). An immunotherapeutic fusion protein, Fn14–TRAIL consists of a portion of Fn14 receptor fused with the TRAIL ligand, which blocks the function of TWEAK. *In vivo* expression of this soluble functional chimera was shown to control the EAE (118). A functional analysis of T-cell response in these mice showed decreased effector Th1 and Th17 responses, and increased number of suppressive Tregs, suggesting that the balance was shifted more toward immune tolerance, which controls the EAE (119). These studies suggest that TWEAK is a very good therapeutic target to control neuroinflammation and autoimmunity. Currently, several TWEAK-targeting therapeutic agents are in clinical trials for autoimmunity and cancer (107).

## CD70–CD27

The CD70 (CD27L, also known as TNFSF7) is a homotrimeric type II transmembrane glycoprotein, and known to express on B cells, T cells, mast cells, NK cells, and activated dendritic cells. It is also expressed on epithelial cells in the thymic medulla. CD70 mainly resides in the endosomal compartment. Its expression is activation-dependent, and controlled by the master transcription regulator of MHC class II gene, CIITA. CD70 binds to its receptor CD27 (TNFRSF7) expressed on CD4^+^ and CD8^+^ T cells, and provides a costimulatory signal, which leads to the proliferation and survival of activated B and T cells. The CD27–CD70 interaction also controls effector and memory responses, and prevents the induction of tolerance (120). Treatment of animals with anti-CD70 antibody suppresses the EAE, possibly by inhibiting the TNF-α production by draining lymph node cells (121). CD70-transgenic mice showed increased numbers of IFN-γ-producing CD4^+^ and CD8^+^ T cells, suggesting that CD70–CD27 signaling controls Th1 (120). It has been reported that CD27^+^ γδ T cells express high levels of IFN-γ and lower levels of IL-17, whereas CD27^−^ γδ T cells express lower levels of IFN-γ and high levels of IL-17, suggesting that CD27 essentially controls the differentiation of γδ T cells, which gives rise to two different effector subsets of γδ T cells (46, 122, 123). The deficiency of CD27 and CD70 in animals leads to significantly increased EAE. In contrast, CD70-Tg mice showed reduced EAE pathology (124, 125). Furthermore, Coquet el al. reported increased Th17 response in CD27^−/−^ and CD70^−/−^, and reduced Th17 in CD70-Tg mice (124). They concluded that this increased Th17 differentiation in CD27^−/−^ and CD70^−/−^ animals were due to increased phosphorylation of the JNK and epigenetic modification at IL-17 locus, suggesting that CD70–CD27 signaling directly controls the Th17 response in CD4^+^ T cells (124). Interestingly, anti-CD70 antibody-mediated blocking of CD70 prevented EAE in SJL/J mice (121), and over-expression of CD70 on the B cells enhanced EAE (126). This is in contrast to what is reported by Coquet et al. (124). These discrepancies might be due to the differences in the mouse strains used or in the cell types where CD70-Tg was over expressed, since over-expression of CD70 in B cells leads to hyper-activation of T cells and a gradual loss of B cells (127). CD70 signaling affects the expression of IL-17F and CCR6 but not other Th17-associated molecules such as RORs, BATF, and IL-23R (124), suggesting that targeting CD27 may be beneficial in controlling effector CD4^+^ T-cell differentiation and its migration into the inflamed tissues during autoimmunity.

## LTβR–LTα

Lymphotoxin alpha (also known as TNFSF1) exists as a secreted homotrimeric molecule produced by lymphocytes. LTα also forms a membrane-anchored heterotrimer with LTβ (Figure 1). LTα homotrimer (LTα_3_) interacts with TNFR-1, TNFR-2, and herpes virus entry mediator (HEVM), whereas the heterotrimer molecule (LTα_1_β_2_) interacts with the LTβR (also known as TNFRSF3) (Figure 1). LTβR is expressed on most cell types, including epithelial cells, endothelial cells, and cells of the myeloid lineages, but not on B and T lymphocytes. LT levels were known to increase in the CNS before the onset of clinical signs of EAE, suggesting a role in the pathogenesis of CNS inflammation. Treatment with LTβR–Ig fusion protein alters the localization of leukocytic infiltration into the CNS and controls the EAE (128). LTα^−/−^ mice have developmental defects in the lymph node formation, and lack Peyer’s patches (129). LTα^−/−^ mice show reduced demyelination and CNS inflammation in MOG_35–55_-induced EAE. However, adoptive transfer of MOG_35–55_-specific wild-type T cells induces EAE (130). This indicates that LT–LTβR signaling mainly regulates the priming phase of myelin-specific T-cell activation and development, but not the actual homing of these auto-reactive cells into the inflamed CNS. In contrast, LTβ^−/−^ mice develop MOG_35–55_-induced EAE, but to a somewhat lesser extent as compared to wild-type animals. These results suggest that LTα plays a very important role in the development of EAE (130). LIGHT (CD258, also known as TNFSF14) is a potent, CD28-independent costimulatory molecule expressed on T cells that are involved in initial T-cell priming and expansion. LIGHT can interact with both LTβR and HEVM. Interaction of LIGHT with HVEM has a costimulatory function, whereas its interaction with LTβR induces apoptosis. LIGHT-deficient mice develop more severe MOG_35–55_-induced EAE due to intensive activation of microglia/macrophages and increased frequency of apoptotic cells within the CNS parenchyma, as compared to wild-type animals (131). Expression of LIGHT on CNS-resident cells rather than myelin-specific CD4^+^ T cells is the most crucial factor in the pathogenesis of EAE. This also suggests that LIGHT expression plays an important role in controlling the activated microglia/macrophages during CNS inflammation. Therefore, selective targeting of LIGHT–HVEM signaling may provide protection against neuroinflammation and CNS autoimmunity. However, the involvement of LIGHT–HVEM interactions with other CNS-resident and infiltrating cells is not well characterized. It would be interesting to study these interactions in different stages of neuroinflammation.

## CD40–CD40L

The CD40 ligand (CD154, also known as TNFSF5) is expressed on activated T cells, and binds to its cognate receptor CD40 (TNFRSF5) on APCs. CD40L–CD40 interactions lead to the activation of B cells, their differentiation into plasma cells, and immunoglobulin class switching. The CD40L–CD40 interactions play a very important role in many autoimmune diseases. The disruption or blocking of this interaction inhibits clinical manifestation and ameliorates the EAE in mice and monkeys (132, 133). In the CNS, various cells, including astrocytes and glial cells express CD40, and expression of this molecule is required for the development of EAE (133, 134). It has been reported that blocking of CD40L–CD40 interaction controls the development of EAE (135, 136). Treatment of animals with remitting EAE in this manner not only controls the EAE, but also inhibits the long-term delayed type hypersensitivity (DTH) response (137). One study has reported that mast cells and astrocytes are localized together in the inflamed CNS, and they have potential to interact with each other via CD40L on mast cells and CD40 on astrocytes (134). *In vitro* studies have shown that this interaction activates astrocytes leading to the secretion of cytokines. These cytokines act in an autocrine manner, and potentiate multiple signaling via the JAK-STAT-1 (Tyr^701^) pathway in the astrocytes (134). Therefore, a contributing signaling event that can enhance the function of infiltrating effector immune cells as well as CNS-resident cells may also promote axonal damage. These reports suggest that CD40–CD40L costimulatory signaling contributes to neuroinflammation and CNS autoimmunity.

## Future Perspective

The growing body of evidence demonstrated that TNF–TNFR interactions are involved in the pathogenesis of EAE and MS. These interactions control the disease outcome by fine-tuning the peripheral immune response as well as interactions between CNS-resident cells and effector immune cells in the CNS. Several reports suggest that the TNFSF–TNFRSF pairs that signal the promotion of inflammation are OX-40–OX40L, sTNFα–TNFR-1, CD27–CD70, 4-1BB–4-1BBL, Fn14–TWEAK, CD40–CD40L, and LT-α/LTβ–LTβR; whereas the ones that show a protective role are tmTNFα–TNFR-2, DR4/DR5–TRAIL, and HVEM–LIGHT. Conflicting results have been reported in the literature about some of the interactions and their importance in CNS autoimmunity. These discrepancies in the results reported by different investigators may be due to the differences in the animal models used or immunization strategies employed to induce the EAE. Furthermore, cross-talks between the signaling induced by various TNFSF–TNFRSF may also contribute to the pathogenesis of disease. Keeping in mind that the combination therapy is central to immunotherapeutic approaches, understanding how and when to block TNFSF–TNFRSF interactions, individually or in combination with other targets, depends on our in-depth knowledge of their expression patterns and molecular mechanisms. Since, there are several TNF superfamily members that have the ability to influence neuroinflammation one way or another, reaching a decision about targeting one or more receptor–ligand pairs at a given time for clinical application requires further investigation. A better understanding of their expression profile, and kinetics of expression, and interactions between TNF ligands and their TNFRs on various CNS residents and infiltrating immune cells at different stages of the disease would help to design better strategies to control neuroinflammation and CNS autoimmunity.

## Conflict of Interest Statement

The authors declare that the research was conducted in the absence of any commercial or financial relationships that could be construed as a potential conflict of interest.

## Abbreviations

APC, antigen-presenting cells; APRIL, a proliferation-inducing ligand; BAFF, B-cell activating factor; BBB, blood–brain barrier; BCMA, B-cell maturation factor; CNS, central nervous system; CTLA-4, cytotoxic T-lymphocyte-associated protein-4; DR, death domain containing receptor; EAE, experimental autoimmune encephalomyelitis; GA, glatiramer acetate; GITR, glucocorticoid-induced TNFR family-related gene; GITRL, glucocorticoid-induced TNFR family-related gene ligand; *gld*, generalized lymphoproliferative disease; GM-CSF, granulocyte–macrophage colony-stimulating factor; HEVM, herpes virus entry mediator; ICAM-1, intercellular adhesion molecule-1; LIGHT, homologous to lymphotoxins, exhibits inducible expression, and competes with HSV glycoprotein D for herpesvirus entry mediator (HVEM), a receptor expressed by T lymphocytes; *lpr*, lymphoproliferative; LTα, lymphotoxin alpha; LTβR, lymphotoxin receptor beta; MMP, matrix metallopeptidase; MOG, myelin oligodendrocyte glycoprotein; MS, multiple sclerosis; RANK, receptor activator of NF-κB; RANKL, receptor activator of NF-κB ligand; TACE, TNF alpha converting enzyme; TACI, transmembrane activator, calcium modulator, and cyclophilin ligand interactor; TNF, tumor necrosis factor; TNFR, TNF receptor; TNFRSF, TNF receptor superfamily; TNFSF, TNF superfamily; TRAF, TNF receptor-associated factor; TRAIL, TNF-related apoptosis-inducing ligand; TRAILR, TNF-related apoptosis-inducing ligand receptor; Treg, regulatory CD4 T cells; TWEAK, TNF-related weak inducer of apoptosis; VCAM-1, vascular cell adhesion molecule-1; VEGI, vascular endothelial cell-growth inhibitor.
